# Cobalt-Incorporated Hydroxyapatite Conditioned Media Promotes In Vitro Scratch Wound Healing and Mesenchymal Stem Cell Migration

**DOI:** 10.3390/jfb16030072

**Published:** 2025-02-20

**Authors:** Weerapat Leelasangsai, Krongrat Thummachot, Puttita Thammasarnsophon, Autcharaporn Srion, Jintamai Suwanprateeb, Somying Patntirapong

**Affiliations:** 1 Department of Implantology, Faculty of Dentistry, Thammasat University, Pathum Thani 12120, Thailand; boomweerapat.l@gmail.com; 2 Faculty of Dentistry, Thammasat University, Pathum Thani 12120, Thailand; krongrat.thu@dome.tu.ac.th (K.T.); puttita.tham@dome.tu.ac.th (P.T.); 3 Biofunctional Materials and Devices Research Group, National Metal and Materials Technology Center (MTEC), National Science and Technology Development Agency (NSTDA), Pathum Thani 12120, Thailand; stks-dcs@nstda.or.th (A.S.); jintamai@mtec.or.th (J.S.); 4 Thammasat University Center of Excellence in Computational Mechanics and Medical Engineering, Thammasat University, Pathum Thani 12120, Thailand; 5 Thammasat University Research Unit in Dental and Bone Substitute Biomaterials, Faculty of Dentistry, Thammasat University, Pathum Thani 12120, Thailand

**Keywords:** cobalt, hydroxyapatite, wound healing, cell migration, mesenchymal stem cell

## Abstract

Cell migration of mesenchymal stem cells (MSCs) is critical for bone healing and remodeling. Cobalt is a well-known hypoxia mimic, which can enhance MSC migration. Therefore, the objective of this study was to investigate the migratory response of MSCs to a developed cobalt-incorporated hydroxyapatite (HACo) material. HACo was fabricated by a simple ion exchange procedure at concentrations ranging from 40 to 8000 μM into disc shape. HACo discs were incubated in the media and conditioned media (CM; HACo_CM_) were collected for MSC culture. HA_CM_ served as a control. MSCs were cultured until reaching 90% confluence before the wound was generated by scraping. Time-lapse imaging of wound migration was monitored, recorded, and assessed. Statistical analysis was performed by one-way ANOVA followed by a Dunnett’s test. The wound area gradually declined from 0 to 40 h for all samples. HACo_CM_ at 40 µM (HACo40_CM_) promoted wound closure at the early period of wound healing. Both HACo40_CM_ and HACo8000_CM_ enhanced the distance and velocity of individual cell migration. However, only HACo40_CM_ affected cell persistence and direction at the early period of cell migration. Exposure to HACo_CM_ accelerated the speed of MSC migration, which is necessary for wound healing. The migratory ability of individual cells could help the rate of wound healing. Therefore, HACo materials may serve as potential biomaterials for enhanced bone healing.

## 1. Introduction 

Bone is a regenerative tissue that continuously remodels throughout its life to preserve structure and function. Osteogenic lineage cells are crucial for bone remodeling. It is essential that osteogenic cells and/or their precursors reach sites that need to be rebuilt. A process in which cells relocate and reach their destination in a given environment to fulfill their function is called cell migration [1]. In the bone healing site, bone mesenchymal stem cells (MSCs) derived either from circulation or resident stem cell pools can be recruited and migrate along osseous surfaces to the previous resorptive sites by transforming growth factor-beta 1 (TGF-β1) [2]. After reaching the site, these cells then start to proliferate, differentiate under the control of insulin-like growth factor 1, and produce bone [3,4]. Failure of osteoblast migration contributes to delayed fracture healing [5]. Thus, the initiation of osteoblast migration could be a key for bone healing. 

Interactions between cells and their extracellular environment can influence cell navigation and migration, which determine healing outcomes [1,6]. The extracellular factors included surface topography and stiffness, tissue-associated growth factors, and cytokines [3,6,7]. One notable external factor is hypoxia, which exists when there is a reduced amount of oxygen in the tissues of the body. Hypoxia is a condition that is reported to stimulate cell migration via hypoxia-inducible factor-1alpha (HIF-1α) activation [8,9]. When the oxygen tension is low, stromal cell-derived factor 1 (SDF-1), C-X-C chemokine receptor type 4 (CXCR4), and HIF-1α expressions are enhanced in MSCs and osteosarcoma cells. Increases in the chemokine SDF-1 and its receptor CXCR4 stimulate migration of cells. Conversely, HIF-1α knockdown reduces SDF-1 and CXCR4 expression, impairing cell migration [8,9]. Changes in chemotaxis behavior exhibit higher cell homing and longer retention to the injury site and thus are beneficial for cell-based therapeutics for wound healing [8,10].

Most adult tissues are hypoxic with an oxygen tension of 2–9%, compared to the ambient tension (21%). Bone is particularly hypoxic relative to other tissues with the oxygen level of 1–6% [11]. Therefore, low oxygen tension is naturally suitable for proliferation, metabolism, and differentiation of MSCs [12]. Two commonly used methods for creating hypoxia in vitro are inducing gas at specific concentrations into an air-tight chamber and using chemical reagents such as cobalt chloride (CoCl_2_) to generate hypoxia mimicry. Co is found in the body as parts of vitamin B12 and co-enzymes [13]. Co supports the use of in vitro hypoxic environments by significantly increasing the expression of stem cell markers and cell migration in stem cells from human exfoliated deciduous teeth [14]. 

Bone remodeling and healing can be impaired and occasionally require bone substitution to restore and rebuild damaged bones. The key strategies of the exogenous material for replacing bone are to have osteoinduction, osteoconduction, and osteointegration properties [15]. The material should allow bone cell migration and bone growth on it, and consequently be replaced with natural bone. Calcium phosphate ceramics have been developed for regeneration approaches in orthopedics and the dental field. From several calcium phosphates, hydroxyapatite (HA) is widely used since it has chemical and structural characteristics similar to the mineral phase of bone and teeth and exhibits excellent osteoconduction and osteoinduction [16]. 

One of the advantages of HA is that its crystalline structure allows foreign ion incorporation. This approach can augment the existing and impart new properties to HA [17]. This makes HA a choice material for bone graft development. We recently attempted to modify HA properties by incorporating Co (HACo) using a simple ion exchange technique [18]. The potential of HACo in enhancing MSC migration remains unexplored. The aim of this study was to examine MSC migratory ability in the presence of Co in HA materials. In pursuit of that goal, the effects of HACo on the migratory ability of whole cell masses and individual cells were investigated. Media incubated with HACo (HACo_CM_) were collected and used to treat MSCs. The whole cell mass was examined for the wound area and the rate of wound healing using Wound_healing_size_tool (Fiji/ImageJ software version 1.53 java 1.8.0). The same software was used to track individual cells for cell front velocity, trajectory, accumulated distance, persistence, and directionality. The addition of Co into HA could improve the material for the use of bone graft in terms of stimulating cell migration. 

## 2. Materials and Methods

### 2.1. Material Fabrication and Conditioned Media Collection

HA powder (Hap-200) was obtained from Taihei Chemical Industrial Co. The sample discs were fabricated according to a previous report [18]. One g of HA powder was soaked in 100 mL of CoCl_2_·6H_2_O (Sigma Aldrich) at concentrations of 40 µM, 400 µM, 4000 µM, and 8000 µM for 1 h at room temperature. The powder was then collected, washed, and dried. A total of 0.28 g of powder was then loaded into a 10 mm diameter stainless steel mold and uniaxially pressed using a bench-top laboratory manual press (Caver, M3853) at a pressure of 2 MPa to obtain the sample disc. The discs were sintered at a temperature of 1100 °C for 6 h using a ramp rate of 5 °C per minute in a chamber furnace (CM, 1610FL). 

The prepared discs were immersed in Dulbecco’s modified Eagle medium (DMEM, Gibco) supplemented with 10% fetal bovine serum (Gibco) and 1% Penicillin–Streptomycin (Gibco) and incubated at 37 °C in the humidified incubator with 5% CO_2_ for 3 days. The conditioned media (CM) were collected and stored for future study. HACo_CM_ prepared at 40 µM, 400 µM, 4000 µM, and 8000 µM were designated as HACo40_CM_, HACo400_CM_, HACo4000_CM_, and HACo8000_CM_, respectively.

### 2.2. Cell Culture and Scratch Wound Assay 

Human bone marrow-derived MSCs were obtained from ATCC (PCS-500-012). Cells at passages 3–5 were used and were maintained in DMEM at 37 °C in the humidified incubator with 5% CO_2_. MSCs were plated in a 24-well plate at a density of 1.5 × 10^4^ cells/cm^2^. The cells were maintained until reaching 90% of confluence before the scratch wound was performed by being scraped with 0.1–10 μL sterile pipette tip (Corning). The tip was placed at the border and perpendicularly to the well surface and was manually drawn from one side through the center to the opposite side of the well. Non-attached cells and debris from scraping were washed with 1× phosphate buffered saline (PBS). Then, HACo_CM_ was added to cells. Cells treated with HA_CM_ served as controls.

### 2.3. Time-Lapse Imaging

After the wounds were initiated, the culture plates were placed under a temperature-controlled chamber set at 37^◦^C with a 5% humidified CO_2_. The incubation chamber is connected to a phase-contrast scanning confocal microscope (Nikon Eclipse Ti; Nikon Instruments, Brighton, MI, USA). A microscope stage is operated with a computer-controlled step motor for monitoring several sites and samples under equal conditions. The exact position of each wound area (center of each well) was defined and set under 10× objective magnification. The microscope was programmed to acquire and record pictures of the wound area every 30 min. Time-lapse imaging was conducted over an 18 h period (time 0 h to time 18 h (t_0_–t_18_)). The culture plates were moved back to the incubator for 4 h, then time-lapse imaging was conducted again for another 18 h period (t_23_–t_40_) with the same setting. These two divided periods were designated the early and late stages of wound healing, respectively. After acquiring the time-lapse images, all images were saved in a TIF file format. Each sample was independently conducted at least 4 times.

### 2.4. Image Processing and Data Analysis 

Image processing and quantitative data analysis were performed using an open-source software Fiji/ImageJ version 1.53 java 1.8.0. Images of the same field were stacked corresponding to their time series starting from t_0_–t_18_ and from t_23_–t_40_. The freely downloaded Plugins “Wound_healing_size_tool” was employed for analysis [19]. Before running the analysis, the right parameters for wound identification were set. A series of wound healing areas at every hour was automatically and sequentially measured using the “Wound Healing Size Stacks Tool” as described by a previous study [19]. The resulting data were obtained and calculated.

Since the initial scratch wound areas were not similar in size, wound areas were converted and are presented as % area relative to the t_0_ area. Wound area (%) was calculated by Equation (1), whereas wound healing closure (%) was calculated by Equation (2) [19]. The rate of wound healing (R_w_) was the rate that a sheet of cells migrated at each time point. The calculation was modified from a previous study [19] as shown in Equation (3).

(1) $$Wound\;area\;\left(\%\right)=\left(\frac{A_{t=∆t}}{A_{t=0}}\right)\times 100\%$$

(2) $$Wound\;closure\;\left(\%\right)=\left(\frac{A_{t=0}-A_{t=∆t}}{A_{t=0}}\right)\times 100\%$$

(3) $$R_{w}=\frac{A_{i}-A_{f}}{t}$$

where *A_t_*_=0_ is the initial wound area, *A_t_*_=Δ*t*_ is the wound area after n hours of the initial scratch, both in μm^2^. *A_i_* is the average of the initial wound area, *A_f_* is the average of the final wound area both in percentage, and *t* is the time span of the assay in hours.

### 2.5. Cell Tracking, Generation of Trajectories, Displacement, and Directionality 

**Scheme 1:**
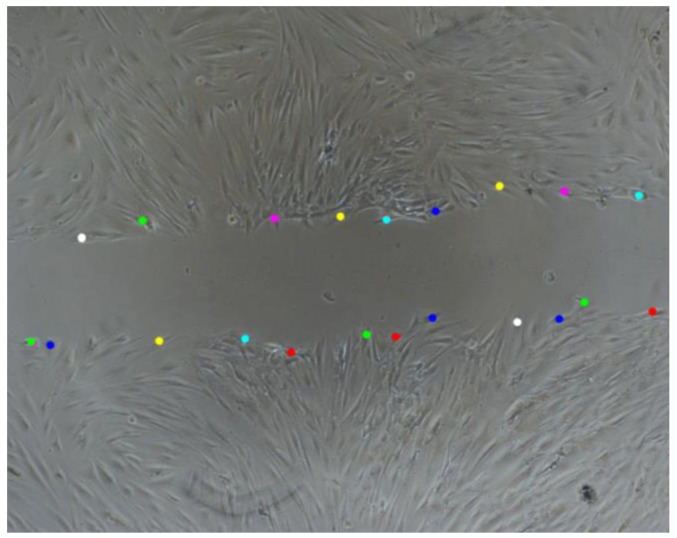
A representative image demonstrates nuclei of individual cells at the wound front marked with different colors.

The leading cells of the wound front were tracked manually by the Manual Tracking function of Fiji/ImageJ. The estimated center of each nucleus of an individual cell was marked (Scheme 1). The marking of each single cell in all time-lapse images from t_0_–t_18_ and t_23_–t_40_ was traced and recorded [20,21]. These steps were repeated in other leading cells. At least 20 cells per sample were measured. Trajectories of cells demonstrating each cell mobility were labeled in different colors. The resulting data (track, slice, x,y axis, distance, and velocity) were recorded. Accumulated distance (µm) and velocity (µm/min) were analyzed. Cell persistence is a directionality ratio calculated by taking the shortest distance between the start and end points divided by the actual traversed path length [6,22]. Displacement and directionality are defined as the distance and direction of a cell moving from its initial position to the final position in a straight line in the x and y axis. 

### 2.6. Statistical Analysis

All statistical analysis was performed by using GraphPad Prism version 5 (GraphPad software). Normality was verified by performing the Shapiro–Wilk test. Data were then analyzed by one-way analysis of variance followed by a Dunnett’s multiple comparison test. Results are presented as means ± SD. All samples were compared with HA_CM_. Statistical significance was assigned as * *p* < 0.05, ** *p* < 0.01, and *** *p* < 0.001. 

## 3. Results

### 3.1. Conditioned Media from HACo Affected Wound Closure In Vitro 

**Figure 1:**
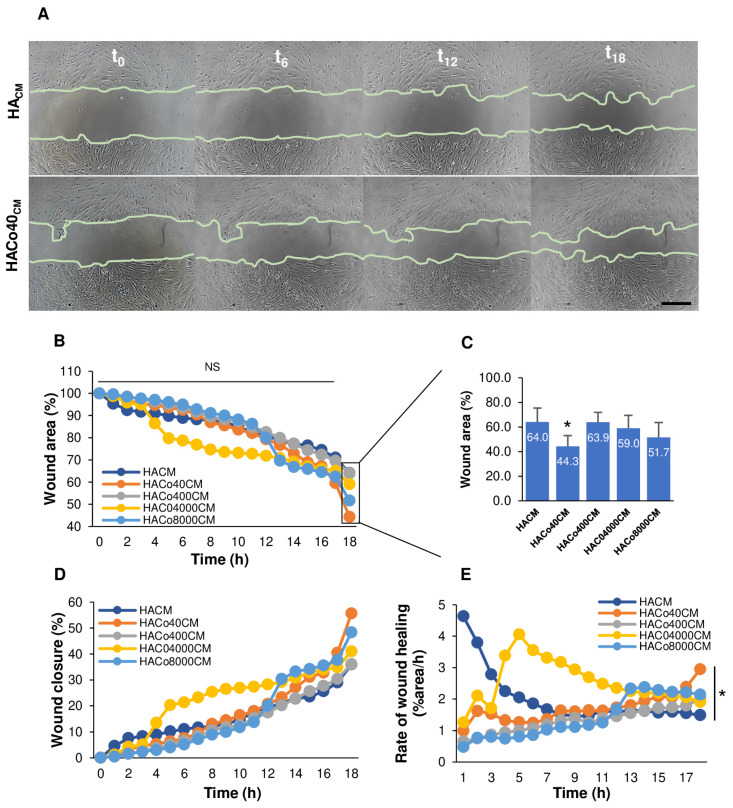
Effects of HACo_CM_ on wound healing of MSCs from t_0_–t_18_. Scratch wounds were created in the middle of the well from left to right (x axis). (**A**) Time-lapse images of wound front. The upper panel is representative images of HA_CM_-treated wound at t_0_, t_6_, t_12_, and t_18_. The lower panel is representative images of HACo40_CM_-treated wound at t_0_, t_6_, t_12_, and t_18_. Light green lines demonstrate the wound margin in each time point. The area between the lines represents the area lacking cells. Scale bar is 200 µm. (**B**) Quantification of the wound area (%) from t_0_ to t_18_. (**C**) A graph presents the mean values of each wound area at t_18_. (**D**) Measurement of wound closure (%) from t_0_ to t_18_. (**E**) The rate of wound healing (%area/h) from t_0_ to t_18_. * *p* < 0.05.

The wound edge migrated as a loose cell sheet toward the wound area. Wound closure progressed continuously throughout the experiment in all samples (Figure 1A). The wound area of the first 18 h period is shown in Figure 1B. The wound area treated with HA_CM_ at t_18_ was 64%. Exposure to HACo40_CM_ significantly reduced the wound area to 44.3% (Figure 1C). Wound closure was shown in Figure 1D. This value is the inverse of wound area. The increase in wound closure demonstrates the reduction in wound area. All treatments were fitted to linear regressions (R^2^ ≥ 0.8934), together with a continuous wound area reduction without significant changes over the period, suggesting a constant rate of wound closure. Figure 1E demonstrates the trend of wound healing speed. The rate of wound healing in cells treated with HA_CM_ dropped in the first 9 h and then was steady until t_18_. The rate of wound healing for HACo40_CM_ and 400_CM_ increased continuously from the start to the end of the experiment. Only at t_18_, cells exposed to HACo40_CM_ significantly enhance the rate of healing compared with those exposed to HA_CM_ (Figure 1E). 

**Figure 2:**
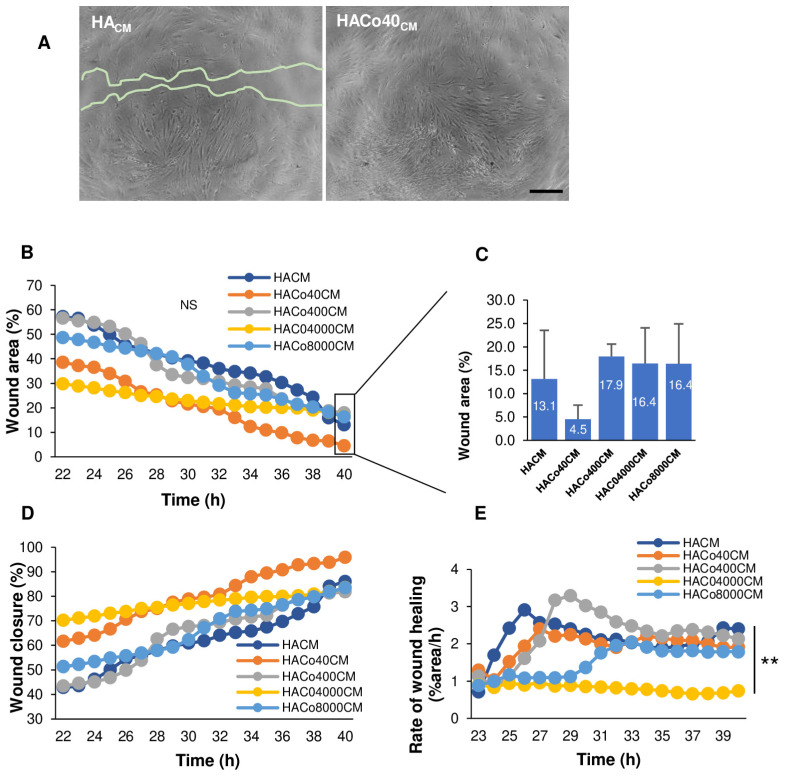
Wound healing of MSCs under HACo_CM_ from t_23_ to t_40_. (**A**) Time-lapse images of wound front at t_40_ after wounding. The left panel is a representative image of HA-treated wound, while the right panel is a representative image of HACo40_CM_-treated wound. Scale bar is 200 µm. (**B**) Quantification of the wound area (%) from t_23_ to t_40_. (**C**) A graph presents the mean values of each wound area at t_40_. (**D**) Measurement of wound closure (%) from t_23_ to t_40_. (**E**) The rate of wound healing (%area/h) from t_23_ to t_40_. ** *p* < 0.01.

Wound healing at t_23_–t_40_ displayed the progression of wound closure. Cells at the wound edge partially migrated as a loose cell sheet. Some cells migrated independently as a single-cell migration. This observation implied that the behavior of individual cell migration differed over time. The final wound area of HACo40_CM_ mostly healed at t_40_ (4.5%), whereas wound areas of HA_CM_, HACo400_CM_, 4000_CM_, and 8000_CM_ were at 13.1–17.9% (Figure 2A–C). Wound closure proceeded constantly (Figure 2D). All treatments were fitted to linear regressions (R^2^ ≥ 0.9463). The rate of wound healing, except for HACo4000_CM_, initially increased and then slightly reduced afterward. HACo4000_CM_ had an even rate of healing at an average value of 1–2% per h throughout the experiment (Figure 2E). Taken together, these results implied that HACo40_CM_ could promote wound closure by reducing wound closure time in vitro. 

**Scheme 2:**
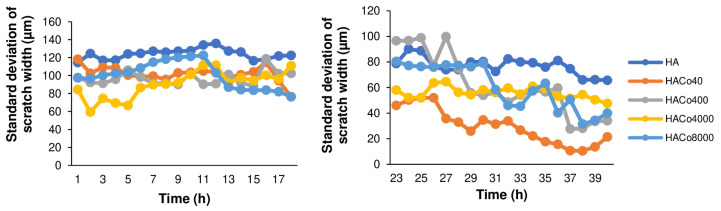
The standard deviation of the scratch width from t_0_ to t_18_ and from t_23_ to t_40_.

In addition, we evaluated the standard deviation of the scratch width according to a previous study [19]. Data showed diverse values (Scheme 2). This value indicates the discrepancy of the wound width at each time point. The heterogeneity of the wound width suggests differences in cell migratory manner [19]; therefore, we further examined individual cell migration to investigate how the cells behaved under different CM.

### 3.2. HACo Conditioned Media Altered Single-Cell Migratory Behaviors

**Figure 3:**
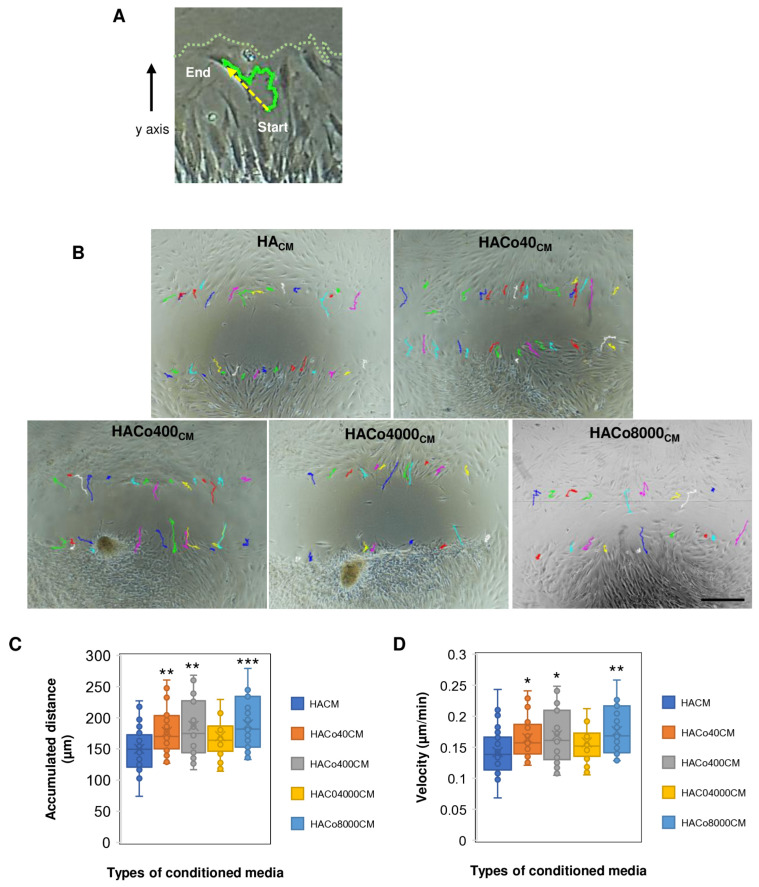
Enhancement of individual cell migration under HACo_CM_ from t_0_ to t_18_. MSC migration was tracked individually. (**A**) Trajectory of a migrating cell from a starting position to an end position is represented by a green line. The displacement of a cell is shown in dashed yellow line with the arrowhead pointing to the end position. A dotted green line labels the wound front. (**B**) Trajectories of representative cells are marked in different colors. Scale bar is 200 µm. (**C**) The accumulated distance of a cell migrated from the starting point to the end point from t_0_ to t_18_. (**D**) The average speed of a cell. The x mark in each box plot represents the mean value. * *p* < 0.05, ** *p* < 0.01, and *** *p* < 0.001.

The trajectory of a migrating cell treated with HA_CM_ was displayed in a green line (Figure 3A). This line was the actual cell path in the 18 h period. Although the cells migrated toward the scratch area, the cells did not move in a straightforward direction but rather in a random direction (x and y axis) (Figure 3A,B). The entire length of the cell transversed in the 18 h period is shown in Figure 3C. The accumulated distance for HA_CM_ was 150 µm. HACo40_CM_, HACo400_CM_, and HACo8000_CM_ significantly increased the distance of cell movement. The velocity of control cells was 0.142 µm/min. The mean velocity of cell migration was increased by HACo_CM_ (Figure 3D).

**Figure 4:**
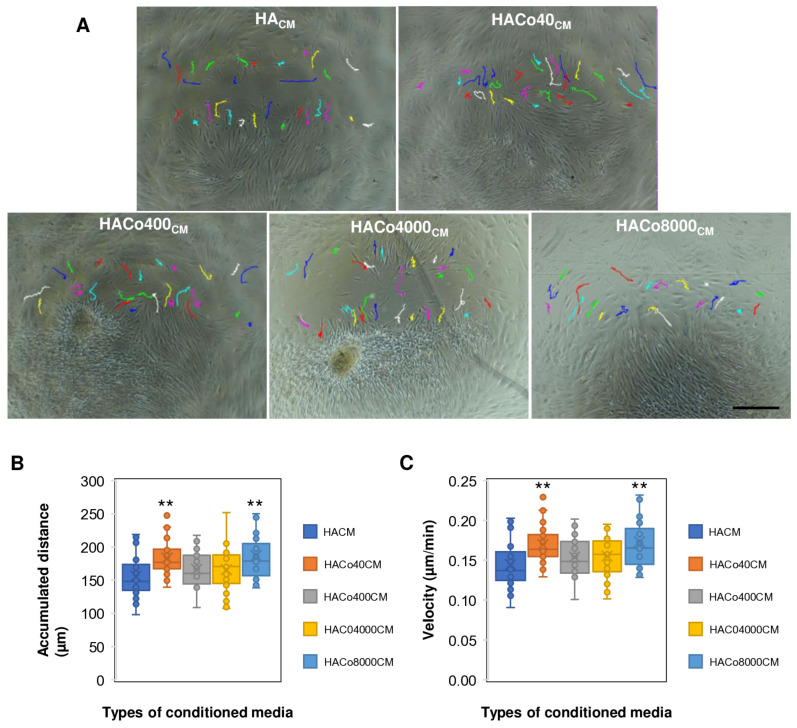
Enhancement of individual cell migration under HACo_CM_ from t_23_ to t_40_. (**A**) Trajectories of representative cells are marked in different colors. The scale bar is 200 µm. (**B**) The accumulated distance of a cell migrated from the starting point to the end point from t_23_ to t_40_. (**C**) The average speed of a cell. The x mark in each box plot represents the mean value. ** *p* < 0.01.

For t_23_–t_40_, the trajectories of selected cells in the sheet and in isolated cells were traced (Figure 4A). The accumulated length for HA_CM_ was 156 µm. The lengths of HACo40_CM_ and HACo8000_CM_ were higher than that of HA_CM_ (Figure 4B). The velocity of control cells was 0.145 µm/min. Treatment with HACo40_CM_ and HACo8000_CM_ significantly increased the velocity of cells compared with HA_CM_ (Figure 4C). These data suggested that HACo_CM_ enhanced individual cell migratory distance and speed. 

**Figure 5:**
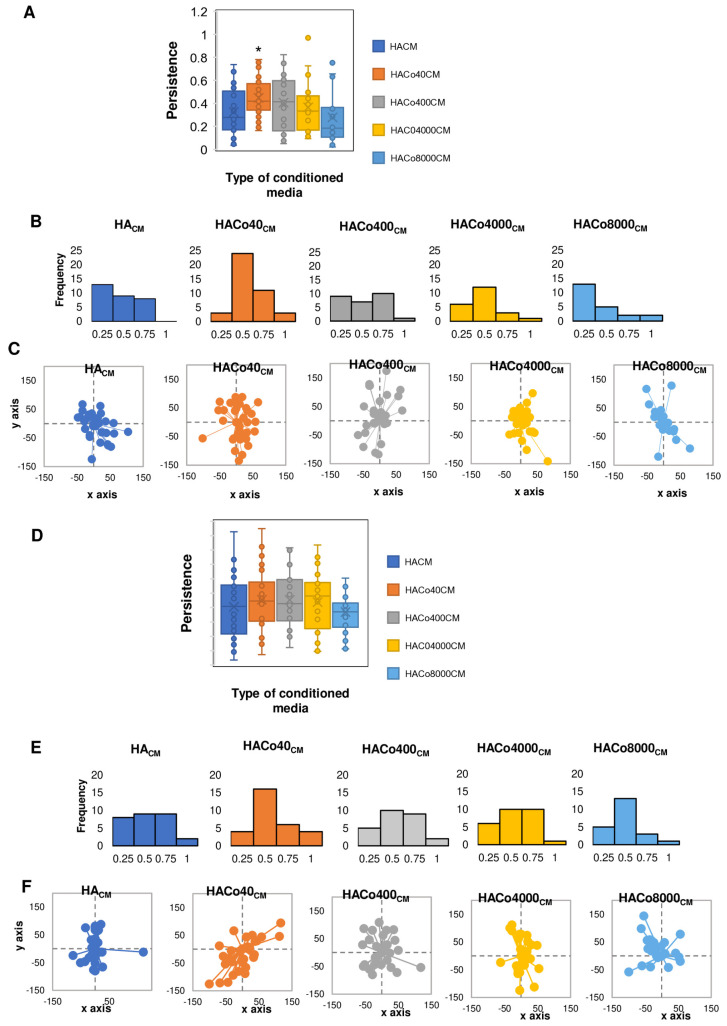
Evaluation of cell directionality and the competency of cell movement under HACo_CM_. (**A**) Persistence of individual cells from t_0_ to t_18_. (**B**) Histogram of cell persistence from t_0_ to t_18_. (**C**) Graphs demonstrate the distribution of each cell displacement from t_0_ to t_18_. The starting position is x = 0, y = 0, while the final position is shown as colored points at x_f_, y_f_. Each line showed the direction of a cell movement in the x and y axis. (**D**) Persistence of individual cells from t_23_ to t_40_. (**E**) Histogram of cell persistence from t_23_ to t_40_. (**F**) Graphs demonstrate the distribution of each cell displacement from t_23_–t_40_. * *p* < 0.05.

Persistence or directionality ratio is a ratio of the distance cell displaced from the starting point to the endpoint (displacement; dashed yellow line in Figure 3A) to the actual length of cell trajectory (green line in Figure 3A) [6,22]. Persistence of HACo40_CM_-treated cells from t_0_ to t_18_ was significantly higher than that of HA_CM_-treated cells (Figure 5A). The persistence of HA_CM_ and HACo8000_CM_-treated cells distributed most in low persistence value, whereas the distribution pattern of HACo40_CM_ and HACo4000_CM_ tended toward a higher persistence value (Figure 5B). The displacement and directionality of the cells from the starting point to the final points were summarized in Figure 5C. The cells treated with HA_CM_ migrated in both the x and y axis of the scratch area. On the contrary, the cells treated with HACo40_CM_ showed more directional preference toward the y axis (Figure 5C). 

No change in persistence among all groups at t_23_–t_40_ was observed (Figure 5D). The distribution pattern of HACo40_CM_ at t_23_–t_40_ was similar to that at t_0_–t_18_ (Figure 5E). The pattern of HACo40_CM_-treated cells altered to a more oblique direction since the cells tended to move to free space between cells (Figure 5F). 

## 4. Discussion

This study investigated the migratory ability of MSCs under various HACo_CM_ using scratch wound assay. After the scratch wound is created, cells move across the denuded area to close the wound. Different cell types migrate at various rates, resulting in different closure times. The time of bone marrow-derived MSCs to entirely fill the scratch wound was more than 40 h. The scratch wound of human corneal epithelial cells closes within a 20 h period post-scraping [23]. The wound closure time of adult human dermal fibroblasts is reported at around 30 h [21]. MSCs exhibited a migration rate of 0.14 µm/min, which is slower compared to other adult human cell types, such as dermal fibroblasts (~0.29 µm/min) [21]. 

We observed that MSCs responded to HACo40_CM_ by reducing the wound area and increasing the rate of wound healing at t_18_. This response accelerated the speed of wound closure from more than 40 h to approximately 40 h. For bone injury, an influx of osteoblast lineage population to the injury site is an important event prior to bone repair/regeneration. MSC migration to the bone-repairing surface is crucial for the bone healing process [24]. Faster migration of MSCs would accelerate bone repair. The addition of CoCl_2_ at 50–100 µM increases the migration of mouse BMSCs and stem cells from human exfoliated deciduous teeth into the scratch wound [14,25]. Therefore, Co addition to HACo40 could be beneficial for the promotion of the early stage of wound healing. 

Epithelial-like cells migrate collectively as coherent sheets to close the wound [26]. However, mesenchymal cells can coordinately move as a mass of individually migrating cells [26,27,28]. Cell migration starts as a loose cell sheet. Due to the lack of strong cell–cell contacts in epithelial cells, the leader cells gradually dissociate from the loose sheet and turn into a cell streaming [21,28]. We speculated that the movement of MSCs was similar to that of human fibroblasts. After the wound is initiated, the leader cells initially have lower speed due to cell–cell association. Later, the cells at the wound edges are no longer contact-inhibited. The cells move toward free space in the center region to cover the wound area. The cell speed gradually increases later on as a result of the dissociation of single cells from the group [21]. 

Since wound healing may depend on the combined efforts of individual cells [6,21], an analysis of a scratch wound from the perspective of individual cells would provide in-depth details of how the wound healing responds to an external stimulus. Cell dissociation from the wound sheet front was promoted by HACo_CM._ Consequently, cell speed and total migratory distance of the leader cells was improved. As cell speed is considered a primary contributor of wound recovery [6,29], an enhancement of individual cell speed by HACo_CM_ could be essential for faster wound closure.

Besides cell speed, other elements that influence the rate of wound closure include cell migratory persistence [6,21] and cell directionality [30]. Both parameters could provide information about the effective movement of the cell. Persistence of individual cells has been shown to correlate with wound closure [6]. Normally, cells do not move from the initial point to the final point in a straight path. A strong difference in persistence value can point to a difference in the direction of the cell movement [31]. A higher value of persistence together with precision in directionality suggests that cells move forward proficiently. Exposure to HACo40_CM_ led to an improvement of cell persistence in the first 18 h. HACo40_CM_-treated cells exhibited better directionality toward the y axis, implying an effective cell movement into the denuded area. Treatment of MSCs with Co could increase individual cell movement efficiency and guide cell migratory direction. 

From all concentrations tested, HACo40_CM_ showed the optimal result in terms of wound healing and individual cell migratory abilities. Although HACo40_CM_, HACo400_CM_, and HACo8000_CM_ promoted individual cell speed and migratory distance of MSCs at early and late stages, only HACo40_CM_ increased cell persistence at the early stage. Interestingly, the persistence result was consistent with the wound healing result. Thus, the persistence of HACo40_CM_-treated cells could determine the wound healing outcome. Our preliminary data found that approximately 5 µM of Co^2+^ ions were released from the sample discs. This concentration was the lowest concentration that induced cell differentiation of primary osteoclasts (data not shown). In this study, this amount stimulated wound healing through individual cell migration. Furthermore, soluble Co^2+^ ions are shown to be dose-dependent in upregulating the chemokine receptor in osteoblast-like cells [32]. While we did not incorporate the Co concentration lower than 40 µM, it is possible that a lower concentration than HACo40_CM_ might exhibit increased cell migration. Cell migration at lower concentrations will fuel future research in the field and has cross-disciplinary implications as well.

Under hypoxic environments such as bone fracture, bone regeneration and fracture healing are enhanced in animal models through the activation of the HIF-1α pathway [33,34]. CoCl_2_ mimics hypoxia by stabilizing HIF-1α and upregulating its mRNA expression [25]. Hypoxic preconditioning of MSC with CoCl_2_ increases HIF-1α expression and C-X-C chemokine receptor type 4 (CXCR4), thus enhancing MSC migratory capacity in vitro. Besides increasing cell migration, hypoxic preconditioning of MSC improves a longer retention time in the ischemic kidney MSC in rats. Increasing recruitment of these cells is associated with the recovery of kidney injury [10]. 

In the injured area, bone healing is suboptimal in terms of cell migration to the wound sites. Modern bone grafts should attempt to overcome the limitation and facilitate the healing process. In this study, Co incorporated into HA improved mesenchymal stem cell migration to the injured area by stimulating scratch wound closure and individual cell movement. This incorporation could be a potential approach for developing a biomaterial for tissue engineering. However, this study is limited to an in vitro study, which was studied on scratch wound assay. Although this assay is simple and mimics migration of cells in vivo, other wound healing techniques should have been performed to strengthen the finding and provide more information. Future studies should incorporate 3D extracellular matrix models and in vivo assays to investigate the effects of HACo on MSC migration. Furthermore, the concentrations of Co released into the media should be examined and tested on other cell types. 

## 5. Conclusions

Migration of cells is an event that underlies tissue remodeling and wound repair. HACo_CM_ stimulated a mass movement of MSCs into the injured area at the early stage of healing. In addition, these CMs affected the behaviors of individual cell movement by increasing cell moving distance and speed as well as directing cell direction toward the wound area. HACo demonstrates promising potential as a biomaterial for enhancing MSC migration and wound healing. Furthermore, designing a biomaterial to treat bone defects should combine the induction effect of cell migration to improve material properties for bone regeneration.

## Data Availability

The original contributions presented in the study are included in the article; further inquiries can be directed to the corresponding author.
